# Development of constructs to measure client satisfaction with pharmacy services in resource-limited settings. A multicenter cross-sectional study

**DOI:** 10.1371/journal.pone.0275089

**Published:** 2022-10-06

**Authors:** Nimona Berhanu, Zewdie Birhanu, Tidenek Mulugeta, Tadesse Gudeta, Belachew Umeta, Gizachew Tilahun

**Affiliations:** 1 School of Pharmacy, Jimma University, Jimma, Ethiopia; 2 Department of Health Behavior, and Society, Jimma University, Jimma, Ethiopia; University of Washington, UNITED STATES

## Abstract

**Background:**

Satisfaction with pharmacy services has many implications, including the degree of interaction with health care providers, the type and quality of service provided, and the extent to which needs and desires are met. This study aimed to identify the dimensions of pharmacy services and quantify client satisfaction with them.

**Methods:**

A quantitative cross-sectional study was employed to guide this study. Data were entered into Epi Data, exported to SPSS 26.0, and analyzed using exploratory factor analysis to identify the underlying dimensions of pharmacy service. The study was conducted between 14th August 2020 and 28th December 2020. For standardization and comparison purposes, items loaded onto each dimension were computed and rescaled, and descriptive statistics were used to summarize the results. Stepwise linear regression was performed to quantify the contribution of each dimension to overall satisfaction and to identify determinant variables for overall satisfaction. A 95% CI, and a P-value of < 0.05 were used for the declaration of statistical significance.

**Results:**

The mean overall satisfaction with pharmacy service was found to be (21.62±6.74)/30. There were eight dimensions of pharmacy service identified, and poor customer satisfaction was recorded for the premises and supply dimensions, with mean satisfaction of (12.08±8.49)/30 and (13.66±10.06)/30, respectively. The highest mean satisfaction was recorded with waiting time (24.24±6.54). Of the emergent dimensions, only four (supply, compassion and care, privacy, and premises) were predictors of overall satisfaction (P<0.05). The supply component was the strongest predictor of overall satisfaction, accounting for 20% of the variance in overall satisfaction. The number of prescribed and dispensed pharmaceuticals, marital status, and gender of participants also predicted overall satisfaction (P<0.05).

**Conclusion:**

The survey uncovered eight underlying aspects of pharmacy services that influence client satisfaction. A significant gap was recorded with premises and supply chain-related components. These dimensions’ contributions to total satisfaction were substantial in terms of practical relevance. As a result, improving the availability of pharmaceuticals and the infrastructure surrounding pharmacy services may enhance consumer satisfaction considerably. Stakeholders must work on addressing supply related and premises difficulties to increase client satisfaction.

## Introduction

In the healthcare industry, satisfaction has many different faces, reflecting the type and quality of service provided by healthcare providers. It also reflects how well service is delivered, and the extent to which the expectations and needs of patients are met [1]. Patient experience encompasses the range of interactions that they have with the health care system, including their care from physicians, nurses, pharmacists, and other staff in the health facility [2]. Pharmacy service is an essential component of the healthcare industry that shares more than half of the resources of health facilities [3]. Client expectations may be a pleasant and welcoming atmosphere for treatment, outstanding service from the providers, the medication they want available, and fair product cost [3, 4]. Client satisfaction has been described in a variety of ways, but the one that appears to have gained the most traction is that satisfaction is a post-choice evaluative judgment of a specific transaction [5]. The high level of satisfaction with pharmacy services means that pharmacies perform their function effectively. Patients are therefore likely to continue obtaining healthcare from health facilities with a high level of customer satisfaction [6].

Despite the fact that many patient satisfaction definitions are ambiguous in pharmaceutical contexts, conceptualizing patient satisfaction using a pharmacy services framework began in the 1970s and made significant contributions to this issue. Prior studies on patient satisfaction with pharmacy services focused mostly on the structure (e.g., pharmacy infrastructure) and the procedure (e.g., prescription filling suitability) [7]. Other researchers added outcome measures including pharmaceutical availability, effectiveness, and quality to assess client satisfaction with pharmacy services [8]. As part of the ongoing effort to develop a comprehensive tool to measure client satisfaction with pharmacy services, other researchers have considered waiting time, pharmacist respect for clients, pharmacy location, and pharmacists’ counseling skills [9]. In terms of identifying pharmacy service dimensions, the study conducted by American researchers identified eight dimensions of pharmacy services: explanation, considerations, technical competence, financial aspect, accessibility, efficacy, product availability, and product quality [8]. Another study conducted in Eastern Michigan identified four dimensions of pharmacy services: communication, physical or emotional comfort, demographics, and location or convenience [10]. Moreover, in 1997 two scholars classified pharmaceutical services based on four patient-satisfaction conceptualizations: performance evaluation (the salient characteristics of the service); dis-confirmation of expectations (the disparity between expectation and experience); user’s emotional reaction to a service and resulting actions (affect-based assessment); and an individual’s appraisal of what is acquired versus what it costs (equity-based assessment) [11]. Early studies in Spain, Qatar, and Nigeria developed two, five, and six dimensions of pharmacy services based on ten, twenty-two, and thirty-five items or questions respectively [12–14].

The evidence clearly shows that client satisfaction scores have a direct implication on the types and quality of services at the bottom line of the healthcare system [15]. Person-centered evaluation of service is among the means of evaluating the quality of overall healthcare operations [16]. Moreover, it is a cost-effective, noninvasive indicator of quality and sustainability of care [17, 18]. In the modern world, the patient is more aware and educated, has access to information, and has expectations from the healthcare system [19]. They could lose confidence in public health facilities if their expectations are not met and if they are unhappy with the services provided in health facilities [20]. Besides, poor perceptions of pharmacy services by clients have been linked to certain patients bypassing public health facilities for an alternative provider and spreading negative word of mouth that can affect potential clients and the facility’s success [21–23].

However, even though the World Health Organization (WHO) and many stakeholders have made significant efforts to make primary healthcare more accessible to the community, clients in Low and Middle-Income Countries (LMICs) still do not have full access to cost-effective, quality-assured pharmaceuticals, a major reason for dissatisfaction among clients [3, 24, 25]. Despite the overwhelming literature on client satisfaction with pharmacy services in Ethiopia [26–29], none of the studies have explored constructs of pharmacy services and quantified client satisfaction with each of them, which could offer insights into the most relevant parameter for improving pharmacy services in such resource-limited environments. A patient satisfaction instrument found to be reliable in one region may not be suitable for another [10]. Therefore, nowadays it is more critical than ever to examine the elements and dimensions of pharmacy services and client satisfaction with them in order to develop operational recommendations and suggestions to improve client access to pharmacy-related services.

## Methods and materials

### Study area

The study was carried out in public health facilities located in Jimma zone from 14^th^ August 2020—28^th^ December, 2020. Jimma zone is one of the 22 zones in the Oromia National Regional State, located at 346 km southwest of the capital, Addis Ababa. Jimma town is the capital of the Jimma zone. The zone has twenty districts and one hundred twenty-five public health facilities at the time of the study. According to the population projection based on the Central Statistics Agency’s (CSA) 2007 census; this zone has a total population of 3,345,112, among which 89.69% are rural inhabitants [30].

### Study design and populations

A facility-based cross-sectional study was employed to guide the study process. Study participants included sampled clients attending outpatient departments (OPDs) of selected health facilities and receiving services from OPD pharmacies during the study period. The study excluded clients with critical illness without a caregiver and those below the age of 18 (without a caregiver). It also excluded health facilities that served as quarantine and treatment facilities for COVID-19.

### Sample size determination

The calculated sample size was 488 consumers visiting health institutions, with 1.5 design effect accounting for stratified and cluster sampling, and a 10% non-response rate, using the single population proportion calculation, n = z^2^ (1-α/2) p (1-p)/d^2^ assuming 74% satisfaction with pharmacy services in public health facilities in Ethiopia [31], 5% marginal error (d), and 95% confidence interval. At the end, 439 clients took part in the study.

### Sampling procedure

**Fig 1:**
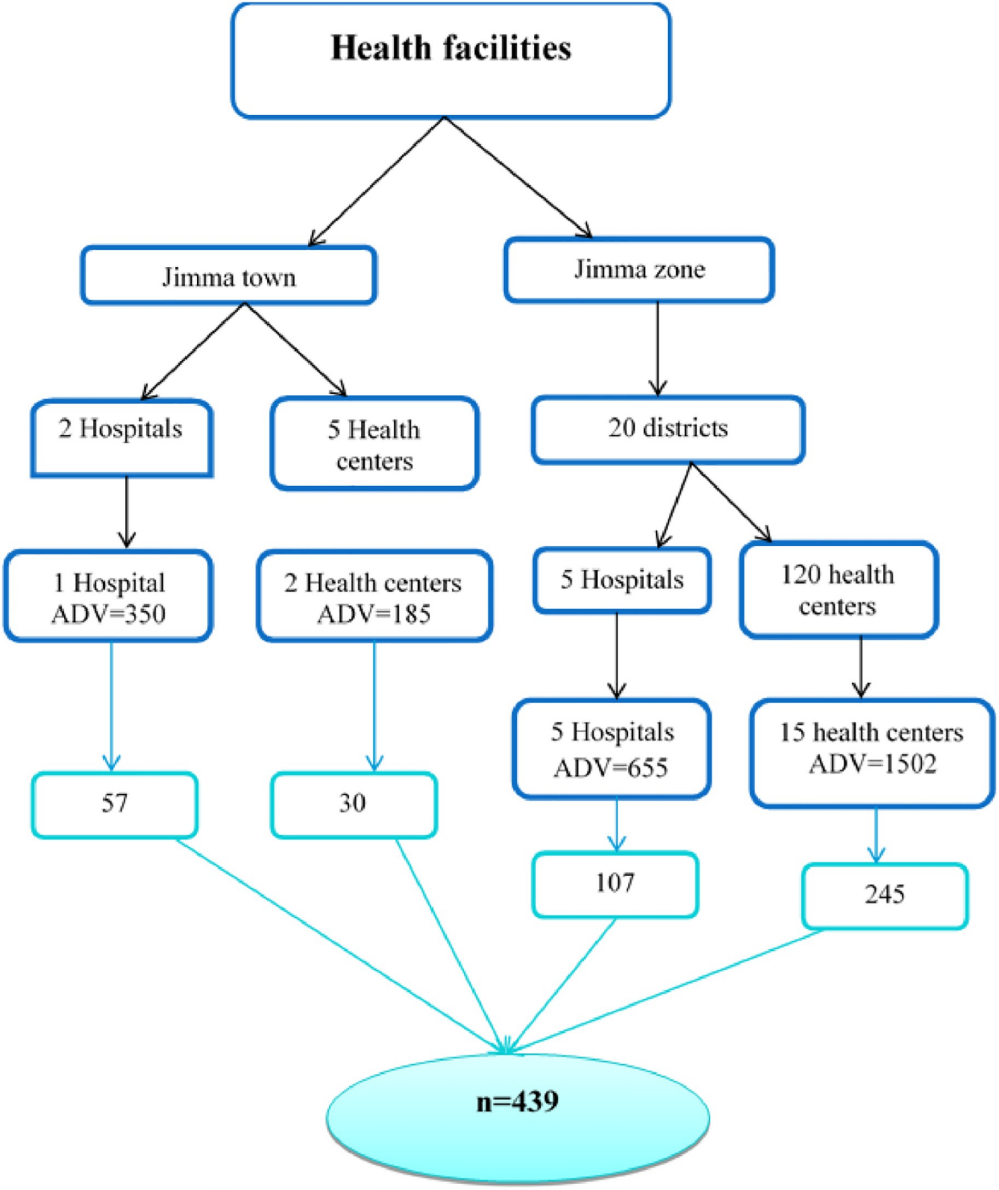
Schematic representation of sampling for health facilities and client, Jimma, August 2020.

The number of health facilities visited in this study was determined based on WHO, and USAID|Deliver recommendations for health facility surveys. According to Logistics Indicator Assessment Tool USIAD|Deliver, at least 15% of health institutions should be assessed in case of resource constraints [32]. Additionally, the WHO advises stratifying healthcare institutions based on their level and managerial authority to guarantee representativeness [33]. Logistics (resources available for the study) were also considered in deciding the number of health facilities included in this study. Considering these factors, twenty-three health facilities (19% of the total public health facilities in the zone) were included in this study. For this study, health facilities were chosen after being stratified by the managing authority (health facilities under Jimma town health office and health facilities under Jimma zone health office). Finally, the sample size was proportionally allocated to each selected health facility considering the three months average daily OPD pharmacy service users, preceding the survey (Fig 1).

### Data collection instrument and data collection

The structured interviewer-delivered questionnaire was constructed after reading relevant literatures [1, 2, 8, 10, 12–14, 26–29, 31, 34–42]. To ensure content validity, the questionnaire was shared for comments with pharmacy and public health professionals. In addition, the last version of the questionnaire was pretested on a random sample of 40 clients in other settings to determine if the questions were clear and how much time was needed to complete them. It took an average of 20 to 25 minutes for one interview to be completed. Finally, the questionnaire was improved based on feedback from participants and data collectors. Generally, it has three main sections, namely, background characteristics of respondents, prescribed pharmaceuticals related information, and satisfaction with pharmacy service items. Satisfaction with pharmacy services was measured using 33 items on a five-point Likert scale ranging from strongly disagree (1) to strongly agree (5) (S1 File). Respondents were interviewed at the point of exit from OPD pharmacies.

### Data processing and analysis

The data from the questionnaire was entered into Epi-Data and then exported to SPSS version 26.00 for analysis (S1 Data). Frequency tables were used to provide information about the respondent’s background characteristics and information on the number of prescribed and dispensed medicines. To evaluate whether the data were suitable for factor analysis, three attributes had to be considered. The three aspects were sample size, correlation matrix factorability, and the Kaiser-Meyer-Olkin (KMO) Measure of Sampling Adequacy or Bartlett’s Test of Sphericity. It is recommended that the sample size should be greater than 100 to execute factor analysis. This survey had 439 clients, which was sufficient for factor analysis. The KMO value should be between 0 and 1, with 0.6 being suggested as the minimum for satisfactory factor analysis, and Bartlett’s test should be significant. The KMO value in our analysis was 0.779, and Bartlett’s p-value was 0.000. Next, exploratory factor analysis using the Varimax rotation method and Kaiser Normalization technique was used to find the underlying dimensions of pharmacy services. The number of significant emergent dimensions maintained in the study was determined using eigenvalue and a scree plot. Only factors with Eigenvalues of 1.0 or above are kept for analysis under this rule. Additionally, satisfaction items that had a significant relationship with the emergent factor (factor loading >0.3) were kept, whereas items that were weakly loaded, double-loaded, or did not load to any factor were removed. For standardization and comparison purposes, the items loaded under each component were added together and rescaled to 30 (0–30) using; **$Y=\frac{\left(X-Xmin\right)n}{Xrange\;}$** formula, where **Y** is the adjusted variable, **X** is the original variable, **Xmin** is the minimum detected value on the original variable, and **Xrange** is the difference between the maximum score, and the minimum score on the original variable, and **n** is the upper limit of the rescaled variable. Following standardization, descriptive statistics such as mean, and median were performed to summarize the findings. After standardization, the Shapiro-Wilk normality test indicated that our data were not normally distributed. For utility and simplicity, items in each factor were classified into ‘Yes’ (agree and strongly agree) and ‘No’ (disagree, strongly disagree, and neutral) and they represented satisfied and unsatisfied clients respectively. Forward stepwise linear regression was used to identify dimensions that independently predicted overall satisfaction, allowing us to explain the contribution of each dimension to overall satisfaction using the weighted value of each dimension (beta-value and R-square). Forward stepwise linear regression was chosen as a method of selecting important variables in order to obtain a simple and easily interpretable model. Since this method chooses the most important variable in the first step, it also enables us to establish the most significant determinant dimension. Similarly, forward stepwise linear regression was performed to identify the effect of other independent variables on the outcome variable. For the declaration of statistical significance, a 95% confidence interval and a level of significance less than 0.05 were used.

### Ethics approval and consent to participate

The study was reviewed and approved by the Institutional Review Board of Jimma University, Institute of Health (Ref. No. IRB000247/20). Respondents were presented with brief information about the study purpose and the process of the study, and consented in written to participate in the survey.

## Results

### Background characteristics of the study participants

The mean age of the respondents was 38.81 ± 12.94 years. Most of the respondents, 306 (67.9%) reported that they could reach (by car) the health facility within less than thirty minutes (Table 1).

**Table 1:** Background characteristics of the respondents, and pharmaceuticals. December 2020 (n = 439).

Background characteristics		Frequency	Percentage
**Sex**	Male	253	57.6
	Female	186	42.4
**Marital status**	Married	366	83.4
	Not married	47	10.7
	Others*	26	5.8
**Age category (years)**	18–25	54	12.3
	26–35	152	34.6
	36–45	116	26.4
	46–55	55	12.5
	> = 56	62	14.1
**Place of residence**	Rural	291	66.3
	Town	148	33.7
**Religion**	Muslim	373	85
	Orthodox	37	8.4
	Protestant	19	4.3
	Others**	10	2.3
**Distance from facility (one-way in minutes)**	< = 30	306	67.9
	31–60	74	16.9
	> = 61	59	13.4
**Occupation**	Government employee	84	19.1
	Merchant	62	14.1
	Farmer	217	49.4
	Student	39	8.9
	Others***	37	8.4
**Service sought for**	Self	312	71.1
	Other person	127	28.9
**Insurance membership**	Yes	269	61.28
	No	170	38.72
**Number of prescribed medicines**	Only 1 medicine prescribed	65	14.8
	2 medicines prescribed	200	45.6
	3 medicines prescribed	128	29.2
	4 and more medicines prescribed	46	10.5
**Number of dispensed medicines**	Only 1 medicine dispensed	179	40.8
	2 medicines dispensed	198	45.1
	3 medicines dispensed	29	6.6
	4 and more medicines dispensed	7	1.7

*Divorced, widowed,

**Catholic, 7^th^ day-Adventist,

***retired, housewife

### Underlying dimensions of pharmacy services

**Fig 2:**
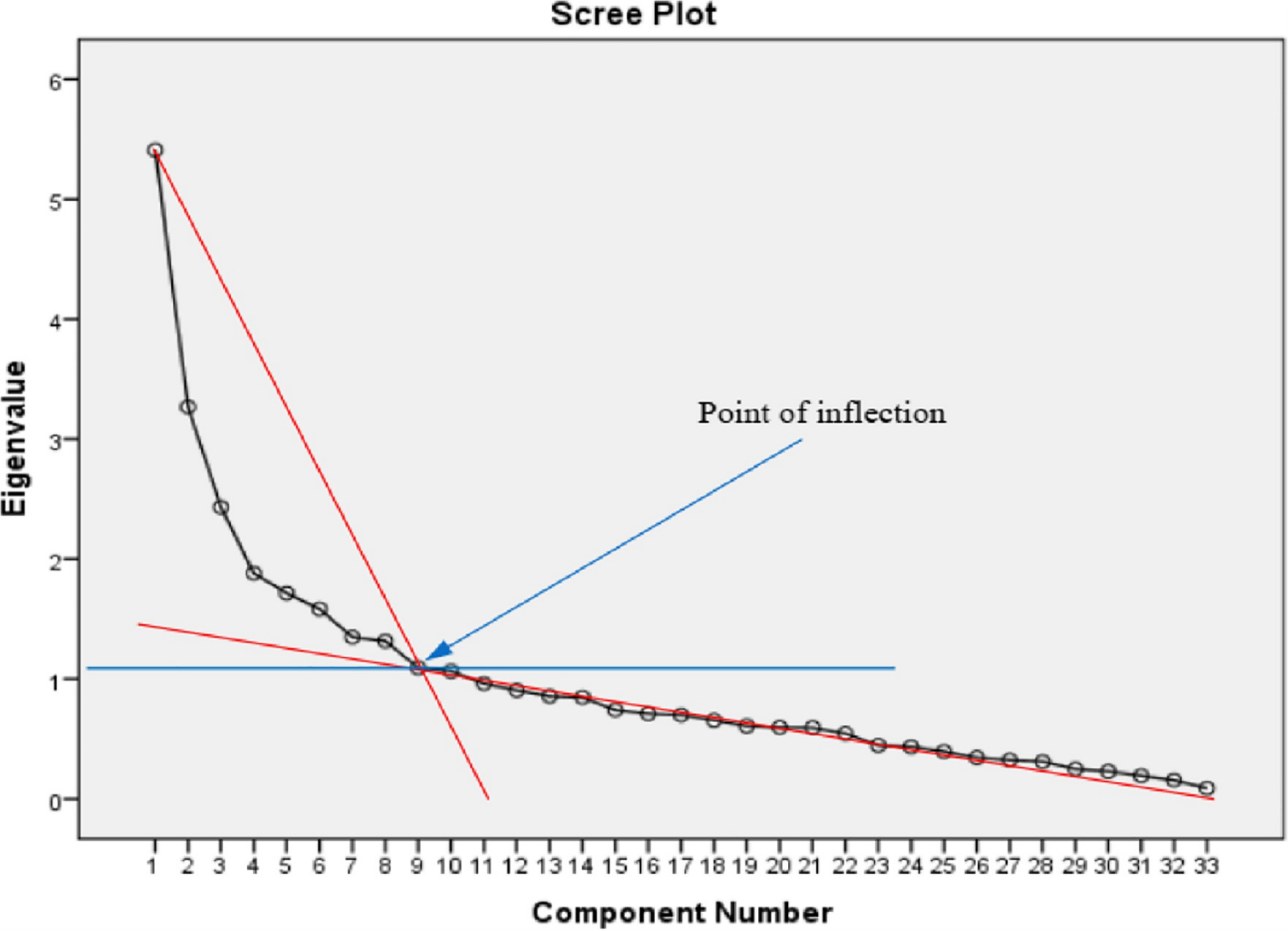
Scree plot showing the number of satisfaction components retained based on eigenvalue, December 2020.

The satisfaction scale items were subjected to factor analysis and the result revealed that the measures produced nine factors (Fig 2), which together explained 63.8% of the variation. One of the nine factors was overall satisfaction, and the other eight were dimensions of pharmacy services. Two items were removed from factor analysis in this analysis due to two reasons: weak factor loading (factor loading 0.30), and loading on two factors, making the final items 31. Among the nine factors, eight components were dimensions of pharmacy service and one component was a compressive contribution of all components (an overall satisfaction reflection after getting all services from the pharmacy). The first component was related to premises (infrastructures of pharmacy in health facilities) and it explained 17.2% of the variance. Another underlying dimension of pharmacy service was related to supply chain aspects of pharmaceuticals and it explained 7.7% of the variance. Other dimensions, namely compassionate, privacy and proper hand-overing, considerations, and advice, waiting time, value for money, and counseling explained 6%, 5.4%, 5.1%, 4.3%, 4.2%, and 3.5% of the variance, respectively. Among the items used to measure client satisfaction, the highest satisfaction level was recorded with dispenser availability at the time of client visit (91.6%) while the lowest satisfaction level was recorded with appropriate labeling of pharmaceuticals before hand-overing to clients (12.8%) (Table 2).

**Table 2:** Emergent dimensions, factor loading and percentage of agreed and strongly agreed respondents with underlying satisfaction items with pharmacy services, December 2020.

Items	Constructs									%A&SA (Yes)	95% CI for Yes (%)
	Prm	Overall	Supp.	Pr&H	Com.	Cons.	Wt.	Vm.	Cng.		
Enough waiting seat in the waiting area	0.85									27.1	22.9–31.3
The waiting area is comfortable and convenient	0.86									25.0	21.0–29.0
The counseling area is comfortable and convenient	0.85									32.6	28.2–37.0
The dispensary room is clean	0.86									31.9	27.5–36.3
The pharmacy room space is adequate	0.84									27.6	23.4–31.8
All the medications prescribed for me are available			0.81							44.4	39.8–49.1
The Pharmacy appears to be stocked with the type of drugs most people need			0.75							29.1	24.9–33.4
I received all the medications from the pharmacy exactly according to the prescription			0.84							36.7	32.2–41.2
Drugs sold at the pharmacy is trusted for their genuineness								0.67		78.6	74.7–82.4
Medication appearance and quality is good								0.69		81.1	77.4–84.5
The amount of out-of-pocket payments for my medicines was fair								0.33		27.5	23.4–31.8
waiting time to get pharmacy service was fair							0.83			79.0	75.2–82.9
Dispenser was available at the time of my visit							0.83			91.6	88.4–93.8
The politeness and interest of dispenser was good					0.62					65.8	61.4–70.3
Dispensers treat the client with dignity and respect					0.83					79.5	75.7–83.3
The language used by Dispenser was easy and understandable					0.45					95.9	94.0–97.3
Dispenser provide service equally for all clients without any favor					0.78					73.1	69.0–77.3
Dispenser asked me important drug and health-related history						0.75				16.9	13.3–20.4
Dispenser mentions information about drug-drug and drug-food interaction						0.63				62.0	56.5–65.6
Dispenser told me about medication precautions and side effects						0.78				25.5	21.4–29.6
The dispenser provides adequate explanation on how to use my drugs									0.75	32.4	28.0–36.7
Dispenser gave me a chance to ask a question on my pharmaceuticals and any ambiguity and any doubts have been resolved									0.75	19.2	15.4–22.8
The dispenser tried to make sure if I understand how to take my medications				0.47						33.3	28.8–37.7
Dispenser gives me the medication with appropriate packaging				0.78						32.4	28.8–36.7
Dispenser gives me the medication with appropriate readable labeling				0.73						12.8	9.6–15.9
The time given for counseling was enough				0.66						35.8	31.3–40.3
The dispenser keeps my privacy				0.45						20.5	16.7–24.3
I was very happy with overall pharmacy services in this health facility		0.78								70.1	65.9–74.5
Next time I am ill, I will come back to this pharmacy		0.81								83.8	80.4–87.3
I was pleased with the way I was treated at the pharmacy		0.78								61	56.5–65.3
If my friends or family are sick I will tell them to come to this health facility		0.80								62.4	57.9–67.0
**% of variance explained (total = 63.8)**	**17.2**	**10.4**	**7.7**	**6.0**	**5.4**	**5.1**	**4.3**	**4.2**	**3.5**		

**Prm**.—premises, **Supp**.—supply, **Com**.—Compassionate, **Pr & H**.—Privacy and proper hand-overing, **Cons**.—Consideration and advice, **Wt**.—waiting time, **Vm**.—Value for money, **Cng**.—Counseling, **% A&SA**—percentage of agreed and strongly agreed

### Item analysis for reliability

An item analysis was conducted to test the reliability of each factor of the pharmacy service construct. The internal consistency of the constructs ranged from 0.62 (counselling) to 0.91 (premises), which is in acceptable to excellent rage (Table 3).

**Table 3:** Cronbach’s Alpha (reliability coefficient) for each construct of pharmacy service, December 2020.

Construct	Items	Reliability coefficients
**Premises**	5	0.91
**Supply**	3	0.84
**Value for money**	3	0.71
**Waiting time**	2	0.64
**Compassionate**	4	0.78
**Considerations and advise**	3	0.68
**Counselling**	2	0.62
**Privacy & proper hand-overing**	5	0.77
**Overall**	4	0.89

### Client satisfaction with dimensions of pharmacy services

**Fig 3:**
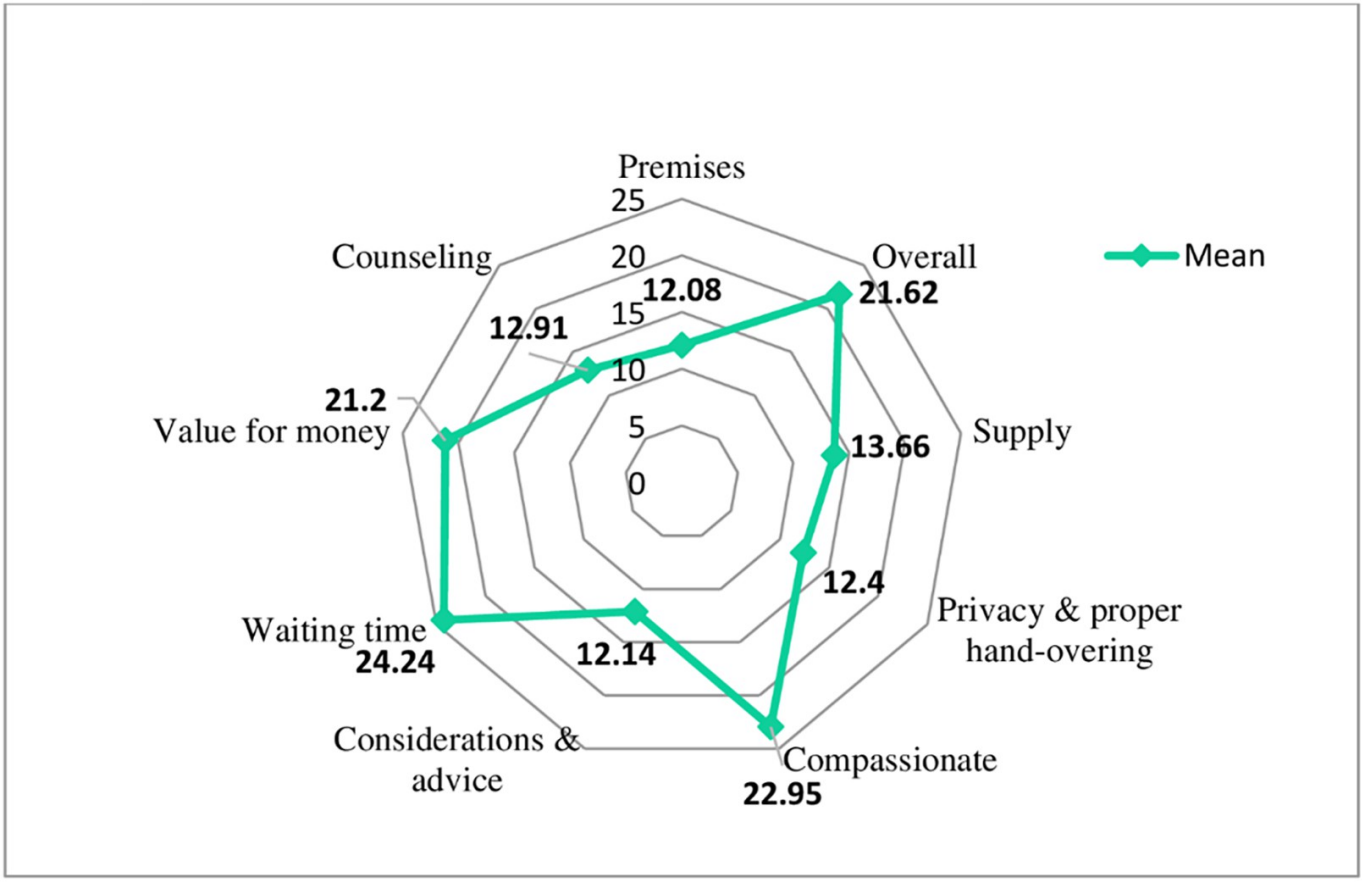
Radar chart, indicating the mean variation for dimensions of client satisfaction, December 2020.

The overall client mean satisfaction was (21.62 ±6.74)/30. The lowest mean satisfaction score was recorded with premises (Mean = (12.08±8.5)/30, and the highest was recorded for waiting time (Mean = (24.24±6.54)/30 (Table 4). The mean satisfaction score near the center of the radar chart shows a low satisfaction level relative to other components. On the other hand, components with the mean score near the edge of the chart show a high level of client satisfaction with the respective dimension of pharmacy service (Fig 3).

**Table 4:** Descriptive statistics of satisfaction constructs (after rescaled), December 2020.

Constructs	Mean (SD)	Median
**Premises**	12.08 (8.49)	9.0
**Overall**	21.62 (6.74)	22.5
**Supply**	13.66 (10.06)	10.0
**Privacy & proper hand-overing**	12.4 (6.36)	12.0
**Compassionate**	22.95 (6.04)	23.08
**Considerations & advice**	12.14 (7.09)	12.5
**Waiting time**	24.24 (6.54)	26.25
**Value for money**	21.2 (5.47)	21.82
**Counseling**	12.91 (6.27)	11.25

### The relative contribution of emerged satisfaction dimensions in overall satisfaction with pharmacy services

Only four pharmacy service dimensions (Supply, Compassionate, Privacy, and Premises) are significant predictors of overall client satisfaction (P<0.05). Furthermore, the supply component accounted for 20% variability in overall client satisfaction with pharmacy services. (Table 5).

**Table 5:** Regression estimate (stepwise linear regression) showing relative contribution of satisfaction dimensions in overall satisfaction, December 2020.

Constructs	R^2^	R^2^ change	Unstandardized Beta	Sig. (P =)	95.0% Confidence Interval for Beta	
					Lower bound	Upper bound
**Supply**	0.200	.200	0.268	0.000	0.22	0.31
**Compassionate**	0.320	.120	0.318	0.000	0.23	0.40
**Privacy**	0.369	.049	0.234	0.000	0.15	0.31
**Premises**	0.376	.006	0.065	0.034	0.01	0.12

### Demographic characteristics and pharmaceuticals factors associated with overall satisfaction with pharmacy services

Compared to unmarried respondents, married study participants were more unsatisfied while males were more satisfied compared to females. The number of prescribed and dispensed medicines was also statistically associated with overall satisfaction (P<0.05) (Table 6).

**Table 6:** Regression estimates (stepwise linear regression) for background characteristics, and pharmaceuticals factors in overall satisfaction, December 2020.

Variable	R^2^	R^2^ change	Unstandardized β coefficients	Sig. (P =)
**Number of prescribed medicine**	0.024	0.024	-2.43	0.000
**Number of dispensed medicine**	0.107	0.083	3.18	0.000
**Married**	0.126	0.019	-2.41	0.003
**Male**	0.140	0.014	1.63	0.007

## Discussion

This study uncovered underlying dimensions of pharmacy services and summarized the level of satisfaction with each of them. We were unable to compare the emerged dimension of pharmacy services and client satisfaction with them with other studies in the same country due to a lack of previous similar studies; therefore, the degree of satisfaction with each item was used to compare with other studies.

The current study identified eight dimensions of pharmacy services, which was much more than the study done in Spain, which discovered two dimensions of pharmacy services (adequacy of services and resources, and interpersonal relationships) [12]. This significant disparity might be attributed to the prior study’s use of only 10 questions to assess pharmacy services. Client expectations and the extent of outpatient pharmaceutical services in the two nations may also contribute to this large disparity.

The study conducted in Qatar identified five constructs of pharmacy service: Promptness, Attitude, Supply, Place, and Teaching. Compared to the current study, the study conducted in Qatar has missed many important elements in pharmacy services like; counseling about drug and food interaction, giving chance for the client to ask questions for any ambiguity, confirming if a client understands the instructions about their medicines, politeness, and respect of pharmacists for the client, and pharmacists’ interest in serving the client equally. The researchers used only 22 items of client satisfaction with pharmacy service to develop pharmacy service constructs, which might be a reason for missing some important items and constructs [13].

Moreover, the study conducted in Nigeria identified six dimensions (attitude of pharmacy personnel, accessibility of pharmacy location, quality and cost of drugs, conducive physical environment availability of prescribed drugs, and timeliness of service delivery [14]. Though most concepts of identified dimensions by the previous study were similar to the current study, still they missed some important dimensions of pharmacy services in resource-limited settings. The scope of OPD pharmacy service in two countries and the number of items used to assess client satisfaction may contribute to the difference in outcome between these studies.

The mean overall (global) satisfaction level of the current study (21.62±6.74/30) was relatively lower than the study conducted in Spain (7.81/10 ~ 23.43/30 [12]. This difference may be due to the method they used to determine client overall satisfaction (they let clients rate their satisfaction level out of ten points). Moreover, a study conducted in Brazil revealed a lower client satisfaction level that the current study (58.4%) [39]. In fact, this might not mean that pharmacy service was better in current study settings than in the pharmacy service settings in Brazil. Because satisfaction is influenced by client expectation and it a post-choice evaluative judgment of service [5].

The study conducted in Ethiopia in multiple hospitals by USAID/SIAPS also revealed a higher overall satisfaction level (74%) than the current study (69.32%) [31]. This discrepancy may be attributed to factors such as the kinds of health facilities included in the two studies as the previous study was done only in hospitals that are far better in resources and infrastructures than health centers. The other likely explanation is the method they used to calculate overall customer satisfaction, as they only used one variable to rate overall customer satisfaction out of 100%.

Unsatisfied consumers have been shown to lose trust in public health services and are more likely to avoid accessing healthcare, particularly public healthcare institutions. Furthermore, low client satisfaction with pharmacy services has been connected to patients skipping public health facilities in favor of a private provider and spreading the unfavorable word of mouth that affects future customers [20, 21]. This was not the case in this survey, since the majority of consumers (83.8%) stated that they would continue to obtain health care from the institution and would recommend that others do likewise (62.4%). In reality, the present statistic does not necessarily imply that consumers were satisfied with the services they provided because the majority of study participants were insurance members who just would not seek health care outside of public health facilities. Other factors, such as the high cost of private facilities and a lack of alternatives, may also play a role in their decision to continue to seek healthcare from the same health facilities.

Supply, compassion, privacy, and premises dimensions of pharmacy service significantly predicted the level of overall (global) satisfaction. The supply dimension was the predominant predictor of overall satisfaction, accounting for 20% of the variability in overall satisfaction. This might be because the majority of study participants were more concerned with the availability of medications than with other services. Compassionate service also plays an important role in improving overall satisfaction. This might be due to the norm of the community strongly supports dignity and respect while seeking services.

Marital status, gender, number of prescribed pharmaceuticals, and the number of dispensed pharmaceuticals were also predictors of client overall satisfaction. Married respondents tend to be less satisfied than unmarried respondents, which was opposite of the study conducted in Qatar that revealed married respondents showed higher satisfaction level than unmarried ones [13]. The current study also revealed that male respondents were more satisfied than females which was the same as a study conducted in Qatar and opposite with the study conducted in Nigeria which revealed females had high satisfaction level than males [13, 14]. The high overall satisfaction level of males in the current study might be due to the difference in expectations from health facilities. Additionally, women are the predominant caretaker for family members and expect more services from public health facilities at an affordable cost.

The study conducted in northwestern Ethiopia and central Ethiopia revealed that there was no statistically significant relationship between sociodemographic characteristics and client satisfaction level [26, 29].

The current study revealed that there was a huge gap in client satisfaction with the premises of studied health facilities. The study conducted in Pakistan also showed an almost similar result with the current study on client satisfaction with this dimension which revealed that only 33% of clients were satisfied with parking facilities [40]. Additionally, the study conducted in Saudi Arabia showed that only 38.4% of clients were satisfied with the counseling area which was higher when compared with the same satisfaction items in the current study (25%) [41]. Moreover, the average satisfaction of clients with premises of pharmacy was 35.1% in the study done in central Ethiopia which was also low but higher than the current study [26]. The reason for the low satisfaction level with this dimension in the current study was due to the poor infrastructure in most public health facilities except in some hospitals in which Auditable Pharmaceuticals Transaction and Services (APTS) was implemented. The building of health centers in the studied area was the same with poor infrastructures, especially with no waiting and counseling area, which made it difficult to keep the privacy of clients. The COVID-19 situation in the country also contributed to the dissatisfaction of clients with this component, as there was a fear of being infected by COVID-19 in crowd conditions while seeking service.

In the current study, only 44.4% of respondents were satisfied with the availability of prescribed pharmaceuticals, which was very poor when compared with a study done in Pakistan, and Tanzania, which revealed that 77.5% and 82.55% of clients were satisfied with the availability of prescribed medications [40, 42]. The higher satisfaction level in the previous two studies was might be due to the difference in economy and health system, which can affect the continuous supply of pharmaceuticals in public health facilities. The study conducted in the eastern part of Ethiopia showed almost similar levels of client satisfaction with the availability of prescribed pharmaceuticals (38.9%) [28]. The previous study was conducted only in two hospitals that can have more resources and system managing capacity to sustain the supply of pharmaceuticals. The relatively low satisfaction level of clients with the supply dimension and its contribution to overall satisfaction suggested that public health facilities were poorly responding to client needs from this perspective.

Additionally, we can learn from this finding that improving the supply of pharmaceutical products to health facilities can boost overall client satisfaction. The major cause for consumers’ displeasure with the supply dimension may be an interruption of pharmaceuticals supply owing to the COVID-19 situation. Other possible reasons are the inability of the country’s sole public pharmaceutical supplier (Ethiopian Pharmaceuticals Supply Agency) to ensure an uninterrupted supply of pharmaceuticals for public facilities, internal facility bureaucracy that hinders simplifying pharmaceutical purchases, and the inability of an insurance organization to complete payment for health facilities so that they could perform pharmaceuticals purchasing on time.

In the current study, clients were least satisfied with appropriate readable labeling on medication while delivering to them (12.8%) which was less than the study conducted in Pakistan (78.8%), United Arab Emirates (43.7%), and central Ethiopia [26, 40, 41]. This suggests that the habit of labeling pharmaceuticals while dispensing was very poor which affected client satisfaction negatively. Moreover, it can lead to misuse of pharmaceuticals, which can have many consequences like drug resistance and resource wastage. This issue may be attributed to a shortage of workforce and a lack of professional interest, which may be caused by poor coordination between the management team and professionals.

In the current study, 65.8% of clients were satisfied with the interest and politeness of dispensers which is somewhat lower than the study conducted in United Arab Emirates which revealed 74.1% of clients were satisfied with the politeness of dispensers [41]. The lower satisfaction level in the current study might be due to the type of health facilities involved in the study as the previous study was conducted in hospitals and the difference in economic development level between countries and enough workers could contribute to the satisfaction level difference. In addition, the study conducted in Eastern Ethiopia revealed that 66.2% of clients were satisfied with the respect and dignity of dispensers for clients which was less than the current study (79.5%) [28]. Another study conducted in central Ethiopia revealed that 89.6%, and 84.4%, of clients were satisfied with the politeness and respect of dispensers, and equity in service delivery for clients respectively which was somewhat higher than the current study [26]. This difference might be due to the number and level of health facilities included in the study as the previous study was limited to only one tertiary care center equipped with many professionals and infrastructure, which missed the variability between different levels of health facilities with different workers and resource.

## Conclusions

Eight dimensions of pharmacy service were identified. The mean of overall satisfaction was high despite the low satisfaction level with some dimensions. Premises and supply components were the two dimensions recorded with low mean satisfaction relative to the other dimensions. This implies that pharmacy services in public health facilities were equipped with poor infrastructure and not ensured a consistent supply of pharmaceutical for their clients. Only four components (supply, compassion, privacy, and premises) of pharmacy service had a statistically significant relationship with overall satisfaction. Among the four components, the supply component was the strongest predictor of overall satisfaction. We can learn from this that ensuring the sustainability of product availability is more important than any other component of pharmacy service in public health facilities in improving client overall satisfaction.

## Supporting information

S1 File (DOCX)

S1 Data (ZIP)

## Abbreviations

APTS: Auditable Pharmaceuticals Transaction and Services
COVID-19: Coronavirus Disease 19
CSA: Central Statistical Agency
LMICs: Low and Middle-Income Countries
ODP: Out Patient Department
USAID: United States Aid for International Development
WHO: World Health Organization
