# Supplementary material for: Development of constructs to measure client satisfaction with pharmacy services in resource-limited settings. A multicenter cross-sectional study
Source: PLoS One. 2022 Oct 6;17(10):e0275089. doi: 10.1371/journal.pone.0275089 (PMC9536595; doi:10.1371/journal.pone.0275089)
Supplement: S1 File — (DOCX) [file pone.0275089.s001.docx]

**Annexes**

**Annex I: Participants information sheet**

**Title of the research: -** Development of constructs to measure client satisfaction with pharmacy services in resource-limited settings. A multicenter cross-sectional study.

**Name of Principal Investigator:** Nimona Berhanu

**Organization/University:** Jimma University

**Purpose of the study:** To identify dimensions of pharmacy services and quantify client satisfaction with them.

**The benefit of the study:** Upon completion of the study, findings will be shared with stakeholders at different levels. Identifying pharmacy service constructs and pressure points on pharmacy services will help stakeholders improve pharmacy services by planning and acting accordingly. Moreover, we will publish the findings so that other interested parties may learn from our research and use it as a reference for similar future research.

**Procedures:** You are invited to participate in this study as a client of the OPD pharmacy of this health facility. You will take part in an interview focused on your background information, information about your medicines, level of your satisfaction with pharmacy services, and overall satisfaction with pharmacy services. These are the types of questions we will ask you, and it will take you about 20 to 25 minutes.

**Risk and Discomforts:** This research poses no risk to participants.

**Benefits:** No incentives will be provided to you for participating in this study. Nevertheless, your honest responses will help us better understand the constructs of pharmacy service, and the level of client satisfaction with each component.

**Confidentiality:** We will maintain the confidentiality of the data we gather for this study. Your name will not appear on the file containing the data about you that will be gathered from the study; instead, it will have a code number. The study team will be the only ones to have access to the data, which will be stored separately in a secure data management file. Your private information won't be shared, not even when the results are reported.

**Right to refuse or withdraw:** You have the choice of participating or not. Without providing a reason, you are free to leave at any time. It will not have any impact on the service you are receiving from the pharmacy if you choose not to participate or withdraw at any moment.

**Whom to contact?** If you have any questions, you can ask now or later. If you wish to ask questions later, you may contact the principal investigator of this research, **Mr. Nimona Berhanu**, Jimma University, e-mail: [nimona2012@yahoo.com](mailto:nimona2012@yahoo.com), phone: +251913241158.

**Annex II: Written informed consent**

I am fully informed about the purpose of the aforementioned study in the language I am able to understand. I understood the purpose of the study entitled “Development of constructs to measure client satisfaction with pharmacy services in resource-limited settings. A multicenter cross-sectional study”. I have also read the information sheet or it has been read to me. In addition, I have been told all the information collected throughout the research process will be kept confidential. I understood my current and future medical services will not be affected if I refused to participate or with draw from the study. I __________, after being fully informed about the detail of this study, hereby gave my consent to participate in this study and approve my agreement with signature.

Client Name ___________________ signature ___________ Date___________

Data collector name _________________signature ___________Date__________

**Annex III. Questionnaire**

Date of Interview_____________ Time Started_____________ Time Finished ___________
Code ____________ Signature of data collecter_____________

| 1. **Socio demographic Characteristics** | | |
| --- | --- | --- |
| 1. Sex | 1. Male 2. Female | |
| 2. Age | ___________Years | |
| 3. Place of Residency | 1. Rural 2. Town | |
| 4. Marital status | 1. Single 2. Married  3. Divorced 4. Widowed | |
| 5. Occupation | 1. Government employee 2. Merchant  3. Farmer 4. Student 4. Other _____ (specify) | |
| 6. Educational level | 1. Can’t read and write 2. Elementary education (Grade1-8)  3. Secondary education (9-12) 4. > Grade 12 | |
| Religion | 1. Muslim 2. Orthodox 3. Protestant 4. Others | |
| 1. Distance from facility | ____________Minutes | |
| 8. Number of visit in this year | 1. First time 2. Second time 3. More than two times | |
| 9. Payment status | 1. Paid in cash 2. Free service 3. Member of community insurance | |
| 10.Community based health insurance membership | 1. Yes 2.No | |
| Service sought for | 1. Self 2. Other person | |
| 1. **Medicine related information (by observation)** | | |
| 1. Number of prescribed medicine | | __________________ |
| 1. Number of dispensed medicine | | __________________ |

| 1. **Study participants satisfaction with pharmacy services** | | | | | |
| --- | --- | --- | --- | --- | --- |
|  | **Strongly disagree (1)** | **Disagree(2)** | **Neutral (3)** | **Agree (4)** | **Strongly agree(5)** |
| 1. Enough waiting seat in the waiting area |  |  |  |  |  |
| 1. It is not difficult to find the pharmacy room in the health facility |  |  |  |  |  |
| 1. The waiting area is comfortable and convenient |  |  |  |  |  |
| 1. The counseling area is comfortable and convenient |  |  |  |  |  |
| 1. The dispensary room is clean |  |  |  |  |  |
| 1. The pharmacy room space is adequate |  |  |  |  |  |
| 1. All the medications prescribed for me are available |  |  |  |  |  |
| 1. The Pharmacy appears to be stocked with the type of drugs most people need |  |  |  |  |  |
| 1. I received all the medications from the pharmacy exactly according to my prescription |  |  |  |  |  |
| 1. The medicines sold at the pharmacy are trusted for their genuineness |  |  |  |  |  |
| 1. Medication appearance and quality is good |  |  |  |  |  |
| 1. The amount of out-of-pocket payments for my medicines was fair |  |  |  |  |  |
| 1. waiting time to get pharmacy service was fair |  |  |  |  |  |
| 1. The dispenser was available at the time of my visit |  |  |  |  |  |
| 1. The politeness and interest of dispenser was good |  |  |  |  |  |
| 1. Dispensers treat the client with dignity and respect |  |  |  |  |  |
| 1. The language used by Dispenser was easy and understandable |  |  |  |  |  |
| 1. The dispenser provide service equally for all client without any favor |  |  |  |  |  |
| 1. The dispenser asked me important medicine and health-related history |  |  |  |  |  |
| 1. The dispenser mentioned information about drug-drug and drug-food interaction |  |  |  |  |  |
| 1. The dispenser told me about medication precautions and side effects |  |  |  |  |  |
| 1. The dispenser provided adequate explanation on how to use my medicines |  |  |  |  |  |
| 1. The dispenser gave me a chance to ask a question on my treatment, and any ambiguity and doubts have been resolved |  |  |  |  |  |
| 1. The dispenser tried to make sure if I understood how to take my medications |  |  |  |  |  |
| 1. The dispenser gave me my medication with appropriate packaging |  |  |  |  |  |
| 1. The dispenser gave me the medication with appropriate readable labeling |  |  |  |  |  |
| 1. The dispenser told me how and where to keep my medicines in my home |  |  |  |  |  |
| 1. The time given for counseling was enough |  |  |  |  |  |
| 1. The dispenser kept my privacy |  |  |  |  |  |
| 1. I was very happy with overall pharmacy services in this health facility |  |  |  |  |  |
| 1. Next time I am ill, I will come back to this pharmacy |  |  |  |  |  |
| 1. I was pleased with the way I was treated at the pharmacy |  |  |  |  |  |
| 1. If my friends or family are sick I will tell them to come to this health facility |  |  |  |  |  |

**We thank you for your participation and honest responses!**
